# Efficacy of the nutritional support team model in the management of patients undergoing total gastrectomy combined with Roux-en-Y anastomosis

**DOI:** 10.12669/pjms.41.1.11112

**Published:** 2025-01

**Authors:** Wenmian Su, Yang Yang, Qian Wang, Wanqiong Zhang

**Affiliations:** 1 Wenmian Su Department of Pharmacy Intravenous Admixture Services, Xingtai Central Hospital, Xingtai, Hebei Province 054000, P.R. China; 2 Yang Yang Department of Gastrointestinal surgery, Xingtai Central Hospital, Xingtai, Hebei Province 054000, P.R. China; 3 Qian Wang Department of Gastrointestinal surgery, Xingtai Central Hospital, Xingtai, Hebei Province 054000, P.R. China; 4 Wanqiong Zhang Department of Gastrointestinal surgery, Xingtai Central Hospital, Xingtai, Hebei Province 054000, P.R. China

**Keywords:** Nutritional support team, Total gastrectomy, Roux-en-Y anastomosis, Nutritional status, Immune function

## Abstract

**Objective:**

To explore the efficacy of the nutritional support team (NST) management model in patients undergoing total gastrectomy combined with Roux-en-Y anastomosis.

**Methods:**

Clinical data of 102 patients who underwent total gastrectomy combined with Roux-en-Y anastomosis in Xingtai Central Hospital from January 2020 to October 2023 were retrospectively collected. Of 102 patients, 53 received the NST model of management (NST group), while 49 were managed by the conventional nutritional support (TN group). The nutritional and immune function status of the two groups before and seven days after the surgery, postoperative rehabilitation status, and the incidence of complications were compared.

**Results:**

After the surgery, levels of albumin and total protein in both groups increased compared to before the surgery and were significantly higher in the NST group compared to the TN group (*P*<0.05). Levels of CD3^+^, CD4^+^, CD4^+^/CD8^+^ in the two groups increased compared to preoperative levels and were significantly higher than the TN group compared to the NST group. In contrast, postoperative CD8^+^ levels decreased and were significantly lower in the NST group (*P*<0.05). The duration of anal exhaust, defecation, and hospitalization in the NST group of patients was shorter (*P*<0.05), but no significance was found in the incidence of complications between the two groups (3.78% *versus* 16.33%) (*P*>0.05).

**Conclusions:**

For patients undergoing total gastrectomy combined with Roux-en-Y anastomosis, the NST nutritional management model can effectively improve nutritional status, enhance immune function, shorten the postoperative recovery process, and the incidence of complications is equivalent to that of patients receiving conventional nutritional support.

## INTRODUCTION

Gastric cancer (GC) is considered a third leading cause of cancer related deaths worldwide.^1^,^2^ GC greatly affects patients’ quality of life and is associated with a heavy socioeconomic and medical burden.^2^,^3^ Surgery remains an important measure for the clinical treatment of early GC patients.^4^,^5^ Total gastrectomy combined with Roux-en-Y anastomosis is the most commonly used surgical approach to ensure treatment effectiveness and improve patients’ quality of life.^5^,^6^ However, due to its invasive nature, surgery can cause varying degrees of damage to the digestive system that may lead to malnutrition and negatively affect patient outcomes.^4^–^6^ Therefore, studies show that strengthening perioperative nutritional management is crucial in GC patients who are undergoing total gastrectomy combined with Roux-en-Y anastomosis.^7^,^8^

The nutritional support team (NST) model of nutritional management is based on continuously monitoring a patient’s nutritional status from admission to discharge. Dietary structure, food intake, and nutritional interventions are dynamically adjusted according to the patient’s dietary intake and nutritional status in order to provide personalized, standardized, and systematic nutrition management.^7^–^9^ Studies show that the NST model is associated with better standardization of nutritional support and may significantly improve patients’ physical health compared to conventional nutritional (TN) interventions.^8^–^10^

However, research on the efficiency of NST model in patients undergoing total gastrectomy combined with Roux-en-Y anastomosis is still scarce. In recent years, our hospital has launched the NST program for GC patients who undergo total gastrectomy combined with Roux-en-Y anastomosis. This study aimed to retrospectively evaluate the intervention effect of the NST model in the treatment of surgical GC patients.

## METHODS

Clinical data of 102 patients who underwent total gastrectomy combined with Roux-en-Y anastomosis in Xingtai Central Hospital from January 2020 to October 2023 were retrospectively selected. Of them, 53 patients received the NST model of nutritional support (NST group), while 49 patients were managed by the conventional TN approach (TN group).

### Ethical Approval:

The hospital ethics committee approved our study on July 8, 2024, No. 2023-KY-51.

### Inclusion criteria:

- Patients met the diagnostic criteria of GC as confirmed by pathological examination and underwent total gastrectomy combined with Roux-en-Y anastomosis.^2^
- Age>18 years old.
- The clinical data of the patients were complete.

### Exclusion criteria:

- Patients with other malignant tumors.
- Patients with kidney, liver, heart, lung and other organ dysfunction.
- Preoperative gastrointestinal dysfunction.
- Patients with mental disorders.
- Patients with chronic gastroenteritis.
- Patients with diseases of immune system, metabolic system and endocrine system.
- Patients with coagulation dysfunction.

### TN model:

Routine diet guidance and health education were given according to the diagnosis, treatment, and nursing routine of a perioperative gastrointestinal malignant tumor. Patients were instructed to fast for 12 hours and not to drink eight hours before the operation. Nasointestinal tube was used routinely during the operation, and nutritional treatment was given after the operation, without early enteral nutrition. The combination of parenteral nutrition and enteral nutrition was routinely implemented 3-5 days after the operation. During nutritional support, attention should be paid to monitoring glucose and lipid metabolism and correcting water and electrolyte disorders. The nutrition support was provided for seven days.

### NST model:

Clinicians, nutritionists, and nurses formed a nutrition intervention group.

*(1) Pre-operation:* The nutritional risk screening tool (nrs2002) was used to screen and evaluate the nutritional status of patients within 24 hours before the operation. For patients with the nrs2002 score of less than three, only regular monitoring and evaluation were given. Patients with the nrs2002 score ≥ 3 were given parenteral nutrition or oral nutrition. The target protein requirement was 1.5~2 g/kg/d, and the target energy was set at 25~30 kcal/kg/d and nasointestinal tube was routinely used during the operation.

*(2) Post-operation:* If there was no contraindication of enteral nutrition, enteral nutrition was implemented from the first day after the operation. On the first day after the operation, 500 ml of glucose and sodium chloride injection were pumped through the nasointestinal tube at a rate of 20-40 ml/h. On the second day after the operation, 500 ml of enteral nutrition suspension was pumped through the nasointestinal tube at the rate of 20-30 ml. On the third day after the operation, 1000-1500 ml of enteral nutrition suspension was pumped through the nasointestinal tube at the rate of 40-60 ml/h. Parenteral nutrition (such as amino acids, glucose, fat emulsion, Calvin, dote, etc.) was implemented for patients with enteral nutrition contraindications. Patients’ tolerance to the nutrient solution was closely monitored and evaluated. Nutrition intervention was adjusted appropriately, the amount of enteral nutrition was gradually increased, and the amount of parenteral nutrition was slowly decreased. The target protein requirement was set at 1.5~2 g/kg/d, and the target energy was set at 25-30 kcal/kg/d. The nutrition support was provided for seven days.

### Data collection:

- Baseline data of patients, including gender, age, body mass index (BMI), disease stage, American Society of anesthesiologists (ASA) classification, and education level.
- Nutritional status seven days after the operation. Levels of serum albumin and total protein were measured by Hitachi 7060 automatic biochemical analyzer (Hitachi; Japan).
- The immune function status before and seven days after the operation. Blood levels of CD3^+^, CD4^+^, CD8^+^, CD4^+^/CD8^+^ were measured by Bricyte E6 flow cytometer (Shenzhen Mindray Biomedical Electronics Co., Ltd.; China).
- Postoperative recovery, including anal exhaust time, anal defecation time, and the length of hospital stay.
- Complications, including vomiting, abdominal distension, diarrhea, and aspiration.

### Statistical Analysis:

All data were analyzed using spss20.0 software (IBM Corp, Armonk, NY, USA) and prism8.0 software (GraphPad, San Diego, USA). The normality of the data was evaluated by the Shapiro-Wilk test. Normal distribution data were expressed as mean ± standard deviation, and a *t-test* was used. The counting data were represented by the number of use cases using the chi-square test. Bilateral *P*<0.05 indicated a statistically significant difference.

## RESULTS

A total of 102 patients (59 males and 43 females) were included. The average age was 55.55±8.19 years (37-71 years). Patients were retrospectively grouped based on the method of nutritional support, with 53 patients in the NST group, and 49 patients in the TN group. There was no significant difference between the two groups in gender, age, BMI, disease stage, ASA classification, and education level (P>0.05) (Table-I).

**Table-I:** Comparison of baseline data between the two groups.

Baseline data	NST group (n=53)	TN group (n=49)	t/χ^2^	P
Male (yes)	28 (52.83)	31 (63.27)	1.137	0286
Age (year)	56.83±7.74	54.16±8.51	1.658	0.100
BMI (kg/m^2^)	23.01±2.68	23.49±2.94	-0.865	0.389
Disease stage [n (%)]				
I	24 (45.28)	20 (40.82)	0.207	0.649
II	29 (54.72)	29 (59.18)		
***ASA grade [n (%)]***				
I	21 (39.62)	18 (36.73)	0.090	0.764
II	32 (60.38)	31 (63.27)		
Education level [n (%)]				
Junior high school and below	32 (60.38)	27 (55.10)	0.291	0.590
High school and above	21 (39.62)	22 (44.90)		

**Fig.1:**
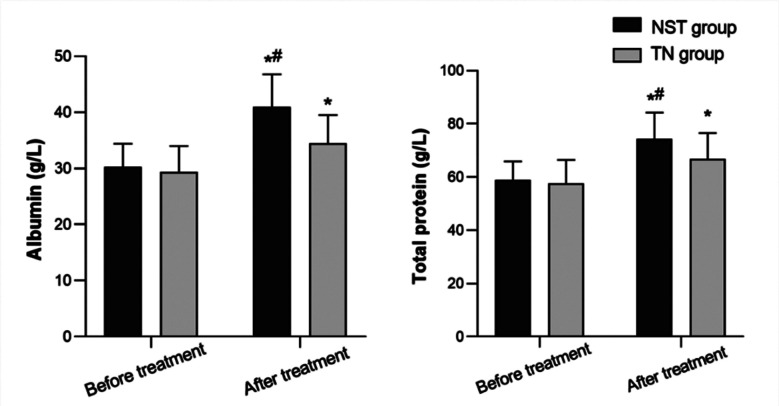
Comparison of nutritional status between the two groups; Compared with before treatment in the same group *P<0.05; Compared with the TN group, ^#^*P<0.05*.

Before the operation, there was no significant difference in the levels of albumin and total protein between the two groups (*P*>0.05). Seven days after the operation, levels of albumin and total protein in the two groups were significantly higher than preoperative, and were significantly higher in the NST group compared to the TN group (*P*<0.05) (Fig.1).

**Table-II:** Comparison of incidence rates of complications between two groups.

Group	n	Vomit	Abdominal distension and diarrhea	Aspiration	Total incidence rate
NST group	53	1 (1.89)	0 (0.00)	1 (.89)	2 (3.78)
TN group	49	2 (4.08)	3 (6.12)	3 (6.12)	8 (16.33)
*χ^2^*					3.229
*P*					0.072

**Fig.2:**
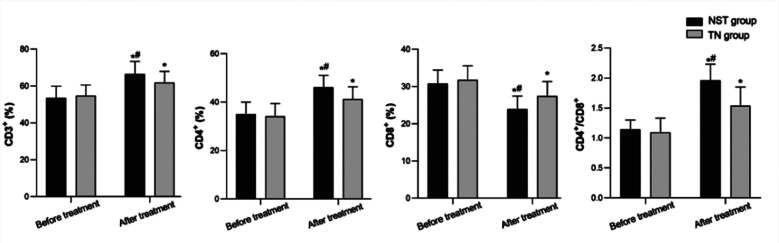
Comparison of immune function between the two groups; Compared with before treatment in the same group. *P<0.05; Compared with the TN group, ^#^*P<0.05*.

**Fig.3:**
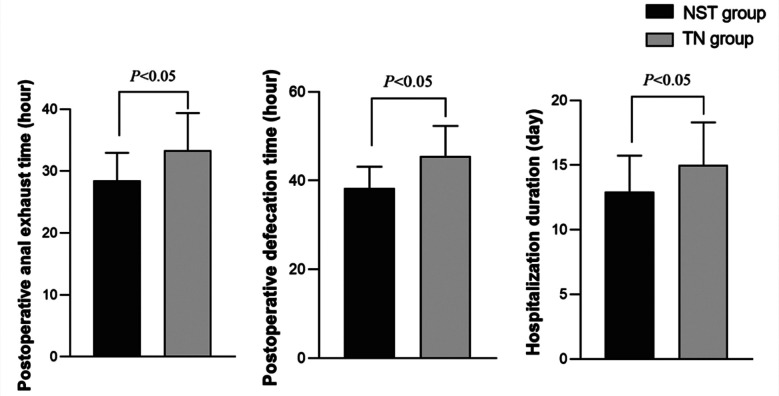
Comparison of postoperative rehabilitation between the two groups.

There was no significant difference in the preoperative levels of CD3^+^, CD4^+^, CD8^+^, CD4^+^/CD8^+^ in both groups (P>0.05). On the 7th day after the operation, levels of CD3^+^, CD4^+^, CD4^+^/CD8^+^ in the two groups were higher than those before the operation, and markedly higher in the NST group compared to the TN group. At the same time, postoperative levels of CD8^+^ were lower than those before the operation and significantly lower in the NST group compared to the TN group (P<0.05) (Fig.2). The postoperative anal exhaust time, anal defecation time, and hospitalization time of the NST group were shorter than those of the TN group (*P*<0.05.) (Fig.3). No significance was found in the incidence of complications between the two groups (3.78% *versus* 16.33%) (*P*>0.05).

## DISCUSSION

Preoperative fasting and delayed eating after the surgery are common practices for total gastrectomy combined with Roux-en-Y anastomosis.^11^ However, in recent years, a series of evidence-based optimization measures, including nutritional management, have been adopted during the perioperative period in order to facilitate rapid postoperative recovery, alleviate the psychological and physiological trauma and stress response of patients, and maintain important and basic physiological functions of the body.^12^,^13^ These measures effectively reduce postoperative adverse reactions, shorten hospital stays, and improve the quality of life of surgical patients.^12^–^14^ The NST model is a systematic, standardized, and individualized quantitative nutritional management model that dynamically adjusts nutritional intervention, dietary structure, and dosage from admission to discharge based on nutritional status, dietary intake, and dietary status.^8^,^13^,^15^ The role of the NST model is increasingly recognized in the field of cancer management, as it can improve overall health and reduce the risk of chronic diseases by reducing inflammation and oxidative stress.^13^,^15^

This study revealed several major findings. Firstly, compared with the TN group, patients who received NST model of management had better nutritional and immune function status seven days after the surgery. Secondly, the time required for postoperative anal ventilation, defecation, and hospitalization was shorter in the NST group. Finally, the incidence of postoperative complications of the NST approach was equivalent to that of conventional nutritional management. Our results are basically consistent with the previous findings. Mulazzani GEG et al.^7^ reported that providing NST during the perioperative period for GC patients can improve the degree of malnutrition, meet the energy and nutritional needs of the body, accelerate recovery, and enhance the quality of life of GC patients.

Chen JS et al.^16^ showed that postoperative levels of CD3^+^, CD4^+^, and CD4+/CD8+ in patients receiving the NST model of nutritional support were higher, while CD8^+^ levels were lower compared to patients who received conventional nutritional support. Additionally, the NST model was associated with lower levels of inflammatory factors such as c-reactive protein (CRP). Jiang XH et al.^17^ also showed that compared to the TN, the NST model considers the specific state of patients to implement corresponding nutritional management. Such an approach is more in line with patients’ physiological and pathological needs. It allows the maintenance of the integrity of the intestinal mucosal barrier, reduces gastrointestinal mucosal damage, and improves nutrient uptake rate. Therefore, the NST approach effectively achieves the goal of improving the body’s nutritional status, which is consistent with our observations.

The TN management protocol does not require a dedicated nutritionist to conduct nutritional risk assessment and nutritional support for patients, and the nutritional status of patients is evaluated by the clinician. However, due to the lack of systematic learning of nutritional management theory, the selection of enteral nutrition has become non-standardized. In contrast, the NST approach is based on the participation of nutrition experts. It can detect nutritional risks and diagnose malnutrition in patients much earlier than the TN approach.^18^,^19^ Timely scientific and standardized nutritional support based on the patient’s condition and nutritional status can effectively improve patient’s nutritional status and body function, ensure functional recovery and improve the prognosis of GC patients after total gastrectomy combined with Roux-en-Y anastomosis.^20^,^21^

### Strength of study:

The strength of this study is that it comprehensively studied the effects of the NST model in terms of nutritional status, immune function, postoperative rehabilitation, and incidence of complication. The findings of the study showed the importance of nutritional support for patients undergoing total gastrectomy. In clinical practices, trained clinicians, nutritionists, and nurses could work together to provide a synergistic nutritional support for the patients to improve treatment outcomes.

### Limitations:

Firstly, since the NST approach does not include pharmacists and economists, we were unable to evaluate the economic costs of implementing an NST model. Secondly, we have not performed follow-up and prognostic analyses. Future studies should validate the efficiency of the NST model for patients’ long-term function and quality of life. Thirdly, this is a single-center retrospective study with a relatively small sample size that only included patients in GC stages-I and II. Further multicenter studies with large patient samples are needed to confirm our observations. Finally, as more and more elderly patients undergo GC surgery, future studies should aim to recruit elderly patients to account for more serious complications that may seriously affect postoperative recovery in this group of GC patients.

## CONCLUSION

For patients undergoing total gastrectomy combined with Roux-en-Y anastomosis, the NST approach of nutritional support may help improve nutritional status, enhance immune function, shorten the postoperative recovery process, and the incidence of complications is equivalent to that of patients receiving conventional nutritional support.

### Authors’ contributions:

**WS:** Concept, study design and manuscript writing.

**YY**, **QW** and **WZ:** Data collection, data analysis, interpretation and critical review.

**WZ:** Was involved in the revision and validation of manuscript

All authors have read, approved the final manuscript and are responsible for the integrity of the study.

## References

[1] Lopez MJ, Carbajal J, Alfaro AL, Saravia LG, Zanabria D, Araujo JM (2023). Characteristics of gastric cancer around the world. Crit Rev Oncol Hematol.

[2] Ogun E, Ekrem UA, Yuksel C, Serdar C, Basceken SI, Umit M (2020). Laparoscopic Gastric Resection for Gastric Cancer:Is Intracorporeal Anastomosis Necessary?. Pak J Med Sci.

[3] Yang WJ, Zhao HP, Yu Y, Wang JH, Guo L, Liu JY (2023). Updates on global epidemiology, risk and prognostic factors of gastric cancer. World J Gastroenterol.

[4] Alakus H, Kaya M, Ozer H, Egilmez HR, Karadayi K (2021). ADAM10 expression in gastric adenocarcinoma:Results of a curative gastrectomy cohort. Pak J Med Sci.

[5] Yin Q, Zhang G, Qie P, Han S, Liu L (2023). Total laparoscopic total gastrectomy and distal esophagectomy combined with reconstruction by transhiatal esophagojejunal Roux-en-y mediastinal anastomosis for Siewert II AEG. J Cardiothorac Surg.

[6] Wang J, Wang Q, Dong J, Yang K, Ji S, Fan Y (2021). Total Laparoscopic Uncut Roux-en-Y for Radical Distal Gastrectomy:An Interim Analysis of a Randomized, Controlled, Clinical Trial. Ann Surg Oncol.

[7] Mulazzani GEG, Corti F, Della Valle S, Di Bartolomeo M (2021). Nutritional Support Indications in Gastroesophageal Cancer Patients:From Perioperative to Palliative Systemic Therapy. A Comprehensive Review of the Last Decade. Nutrients.

[8] Jiang Z, Lin H, Huang J, Sharma R, Lu Y, Zheng J (2024). Lack of clinical benefit from preoperative short-term parenteral nutrition on the clinical prognosis of patients treated with radical gastrectomy for gastric cancer:a two-center retrospective study based on propensity score matching analysis. J Gastrointest Oncol.

[9] Sakai T, Maeda K, Wakabayashi H, Nishioka S, Seki H (2017). Nutrition Support Team Intervention Improves Activities of Daily Living in Older Patients Undergoing In-Patient Rehabilitation in Japan:A Retrospective Cohort Study. J Nutr Gerontol Geriatr.

[10] Guo ZQ, Yu JM, Li W, Fu ZM, Lin Y, Shi YY (2020). Survey and analysis of the nutritional status in hospitalized patients with malignant gastric tumors and its influence on the quality of life. Support Care Cancer.

[11] Resanovic A, Randjelovic T, Resanovic V, Toskovic B (2018). Double Tract vs. Roux-en-Y Reconstruction in the treatment of Gastric Cancer. Pak J Med Sci.

[12] Ida S, Hiki N, Cho H, Sakamaki K, Ito S, Fujitani K (2017). Randomized clinical trial comparing standard diet with perioperative oral immunonutrition in total gastrectomy for gastric cancer. Br J Surg.

[13] Huang L, Hu Y, Chen J (2024). Effectiveness of an ERAS-based exercise-nutrition management model in enhancing postoperative recovery for thoracoscopic radical resection of lung cancer:A randomized controlled trial. Medicine (Baltimore).

[14] Ma J, Li Z, Chen Y, Zhang Y, Wang Q, Yan G (2024). Perioperative nutrition management in patients with spinal tuberculosis taking ERAS measures. Asia Pac J Clin Nutr.

[15] Xu R, Chen XD, Ding Z (2022). Perioperative nutrition management for gastric cancer. Nutrition.

[16] Chen J, Zou L, Sun W, Zhou J, He Q (2022). The effects of nutritional support team intervention on postoperative immune function, nutritional statuses, inflammatory responses, clinical outcomes of elderly patients with gastric cancer. BMC Surg.

[17] Jiang XH, Chen XJ, Chen S, Chen JM, Yuan XH, Lin YJ (2022). Compliance with Oral Nutritional Supplementation among Gastric Cancer Patients at Nutritional Risk:A Cross-Sectional Study. Nutr Cancer.

[18] Lobo DN, Gianotti L, Adiamah A, Barazzoni R, Deutz NEP, Dhatariya K (2020). Perioperative nutrition:Recommendations from the ESPEN expert group. Clin Nutr.

[19] Hsu PI, Chuah SK, Lin JT, Huang SW, Lo JC, Rau KM (2021). Taiwan nutritional consensus on the nutrition management for gastric cancer patients receiving gastrectomy. J Formos Med Assoc.

[20] Gustafsson UO, Ljungqvist O (2011). Perioperative nutritional management in digestive tract surgery. Curr Opin Clin Nutr Metab Care.

[21] Cheng Y, Zhang J, Zhang L, Wu J, Zhan Z (2018). Enteral immunonutrition versus enteral nutrition for gastric cancer patients undergoing a total gastrectomy:a systematic review and meta-analysis. BMC Gastroenterol.

